# ﻿Two new species of Metapocyrtus (Orthocyrtus) Heller, 1912 (Coleoptera, Curculionidae, Entiminae) from southern Mindanao, Philippines, with ecological notes

**DOI:** 10.3897/zookeys.1116.83236

**Published:** 2022-08-09

**Authors:** Analyn A. Cabras, Rizalyn Cudera, Joelyn Mamon, Milton Norman D. Medina

**Affiliations:** 1 Coleoptera Research Center, Institute of Biodiversity and Environment, University of Mindanao, Davao City, 8000, Philippines University of Mindanao Davao Philippines; 2 Sultan Kudarat State University, EJC Montilla, Tacurong City, Sultan Kudarat, 9800, Philippines Sultan Kudarat State University Tacurong Philippines

**Keywords:** Biodiversity, change of placement, Cotabato, new species, taxonomy, weevils

## Abstract

Two new species of the genus MetapocyrtusHeller, 1912,subgenusOrthocyrtus Heller, 1912 are described and illustrated from southern Mindanao, Philippines: M. (O.) melibengoy**sp. nov.** and M. (O.) flomlok**sp. nov.** Another two species were transferred from the subgenus Artapocyrtus Heller, 1912 to *Orthocyrtus*, namely, M. (O.) willietorresi Cabras & Medina, 2018 and M. (O.) villalobosaePatano et al., 2021. Ecological notes are provided.

## ﻿Introduction

Members of the subgenus Orthocyrtus Heller, 1912 (genus *Metapocyrtus* Heller, 1912) are among the most notable of the tribe Pachyrhynchini for their conspicuously large size. The subgenus is currently recognized as endemic to the Philippines and is distributed all over the archipelago. Up to the present, the subgeneric division of *Metapocyrtus* is uncertain and badly needs revision. However, members of *Orthocyrtus* have distinct and stable characters that distinguish them from other subgenera: 1) large species, with a few exceptions; 2) rostrum of medium length, dorsally straight, mostly in a plane with the front (exceptionally slightly concave) and at the base, the sides are rectangularly declined; 3) female, with a few exceptions, without any secondary sexual structural characters aside from a stouter form, and similar to the male; (Schultze 1925; Cabras et al. 2018). The biology and ecology of the genus remain understudied; however, members of the subgenus can be found in a range of habitats which include lowland coconut farmlands, mid-elevation between 300–800 m in mixed secondary forest, as well as old-growth primary and secondary forests (Cabras et al. 2018; Cabras and Medina 2019; Cabras et al. 2021a, b).

In the past two years, two species belonging to *Orthocyrtus* were collected from southern Mindanao and found to be new to science. In this paper, the two new species are described and illustrated. Short notes on their ecology are provided. Another two species from the subgenus Artapocyrtus Heller, 1912 were transferred to *Orthocyrtus*, namely, M. (O.) willietorresi Cabras & Medina, 2018 and M. (O.) villalobosaePatano et al., 2021.

## ﻿Materials and methods

The specimens deposited in the University of Mindanao Coleoptera Research Center were collected by sheet beating and handpicking and killed in vials with ethyl acetate. Morphological characters were observed under Luxeo 4D and Nikon SMZ745T stereomicroscopes. The treatment of the genitals follows Yoshitake (2011). Anatomical parts of the female genitalia are not illustrated as very little of the chitinous structures are used to identify and characterize different species of Pachyrhynchini (Cabras et al. 2021a). Images of the habitus were taken using a Nikon D5300 digital camera with a Sigma 18–250 macro lens. Images were stacked and processed using a licensed version of Helicon Focus 6.7.0, then contrast adjusted in Photoshop CS6 Portable software. Label data are indicated verbatim.

Abbreviations and symbols mentioned in this paper are abbreviated as follows:

/ different lines;

// different labels;

**LB** body length, from the apical margin of pronotum to the apex of elytra;

**LR** length of rostrum;

**LP** pronotal length, from the base to apex along the midline;

**LE** elytral length, from the level of the basal margins to the apex of elytra;

**WR** maximum width across the rostrum;

**WP** maximum width across the pronotum;

**WE** maximum width across the elytra.

Comparative materials and specimens used in the study are deposited in the following institutional collections:

**PNM**Philippine National Museum of Natural History, Manila, Philippines;

**SKSUABC** Sultan Kudarat State University ACCESS Biological Collection, Tacurong, Philippines;

**SMTD** Senckenberg Natural History Collections, Dresden, Germany;

**UMCRC** University of Mindanao Coleoptera Research Center, Davao City Philippines.

## ﻿Taxonomy

### ﻿ Metapocyrtus (Orthocyrtus) melibengoy

Taxon classificationAnimaliaColeopteraCurculionidae

Cabras & Medina sp. nov.

54AB5D36-2A12-5B6D-9B6F-D0430D6D17FB

https://zoobank.org/D82A7BCD-3E58-441F-B651-CBAF006469CB

Figs 1–4

#### Type material.

**Figures 1–4.**
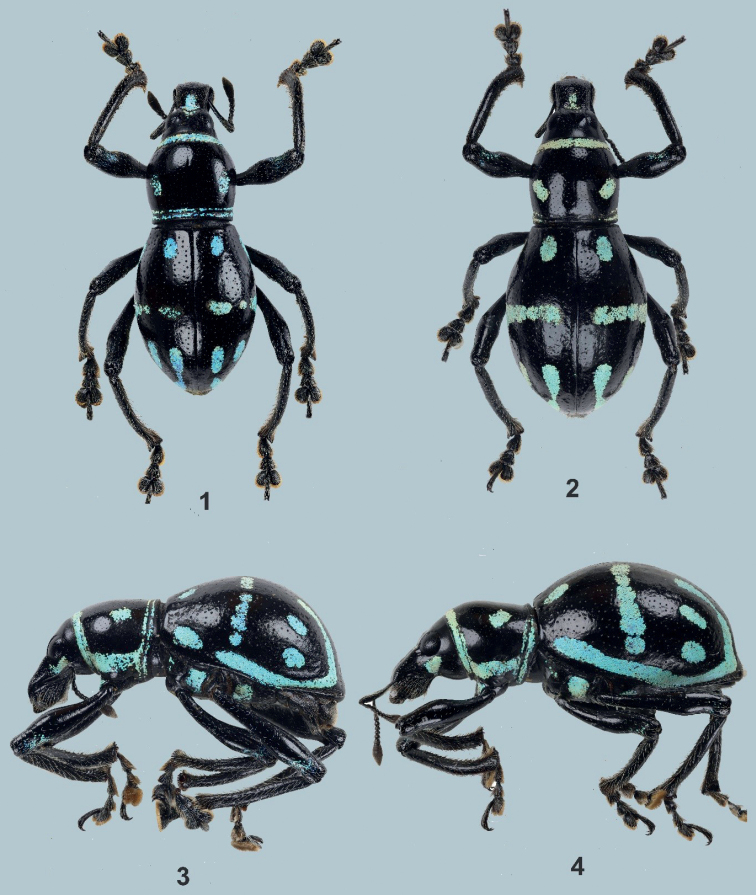
Metapocyrtus (Orthocyrtus) melibengoy sp. nov. **1** male holotype, dorsal view **2** female, dorsal view **3** male, lateral view **4** female, lateral view.

***Holotype*** (Figs 1, 3), male: Philippines - Mindanao / Lake Holon / South Cotabato / October, 2021 / coll. Cabras (typed on white card) // HOLOTYPE male / Metapocyrtus (Orthocyrtus) melibengoy / CABRAS & MEDINA, 2022 (typed on red card). Presently in UMCRC, will be deposited in PNM. ***Paratypes*** (2♂♂, 3♀♀): same data as holotype; all in UMCRC; (3♀♀): Philippines - Mindanao / Lake Holon / South Cotabato / October, 2021 / coll. Mamon, all in SKSUABC. All paratypes with additional red label: PARATYPE / Metapocyrtus (Orthocyrtus) melibengoy / CABRAS & MEDINA, 2022.

#### Diagnosis.

Metapocyrtus (Orthocyrtus) melibengoy sp. nov. is related to Metapocyrtus (Orthocyrtus) lanusinus Schultze, 1922 but differs in its pronotal and elytral scaly markings. Metapocyrtus (Orthocyrtus) melibengoy sp. nov. has two small spots on each side of the disc of its pronotum, and each elytron with one small subbasal spot near the suture, one median stripe from the suture to the lateral side, two subapical spots, a short post-median stripe at stria III, and a long stripe along the lateral margin, confluent with the post-median stripe.

#### Description.

**Male.** Dimensions: LB: 9.2–10.4 mm (holotype 10 mm). LR: 2.0–2.5 mm (2.0 mm). WR: 1.4–1.7 mm (1.4 mm). LP: 3.1–3.6 mm (3.1 mm). WP: 3.6–4.0 mm (3.6 mm). LE: 6.1–6.8 mm (6.1 mm). WE: 5.5–5.7 mm (5.7 mm). *N* = 3.

Integument black. Body surface, rostrum, head, and underside moderately shiny. ***Head*** finely punctured on dorsum with sparse and very minute pubescence, dorsal surface with a scaly patch of metallic pale-yellow ochre and turquoise round scales near the transverse groove; lateroventral side below the eye with a semi-elliptical scaly patch of metallic pale-yellow-ochre and turquoise round scales interspersed with adpressed metallic bluish piliform scales; forehead between eyes slightly depressed; eyes medium-sized and feebly convex. ***Rostrum*** weakly rugose and coarsely punctured on basal 2/3 and finely punctured on apical third, longer than wide (LR/WR:2/1.4mm), dorsum covered with sub-adpressed brownish setae, with large subelongate scaly patch of overlapping light-yellow-ochre, turquoise and bluish round scales on basal half, lateral surface with minute subadpressed brownish setae interspersed with long suberect whitish setae, ventral surface with long suberect whitish setae; transverse basal groove distinct; longitudinal groove along midline distinct and forms a shallow concavity; dorsum almost flattish dorsally and apex weakly convex; lateral sides with moderately widened apicad. Antennal scape and funicle almost the same length, scape reaching slightly behind the hind margin of eye, covered with subadpressed fine light-colored setae, and funicle with suberect brownish setae. Funicular segments I and II are almost of the same length, three times longer than wide; segments III–VII nearly as long as wide; club sub-ellipsoidal, nearly 3 times longer than wide. ***Prothorax*** subglobular, wider than long (LP/WP:3.1/3.6 mm), finely punctured with minute pubescence, widest at middle, weakly convex on dorsal surface, dorsal contour highest point on basal ¼. Prothorax with the following scaly markings of metallic, light-yellow ochre and turquoise, round scales: a) stripe at the anterior margin, b) two small subcircular spots on each side of discs, c) stripe at the posterior margin, and d) slightly broader stripe before the coxa confluent with the anterior and posterior marginal stripes. ***Elytra*** ovate (LE/WE:6.1/5.7 mm), moderately wider and nearly twice longer than prothorax (WE/WP: 5.7/3.6 mm, LE/LP: 6.1/3.1 mm), distinctly and randomly punctured with very minute pubescence, dorsum strongly convex, dorsal contour highest before the middle, lateral contour evenly arcuate, widest at middle, apex quite rounded with sparse, white, fine setae. Each elytron with the following scaly markings of pale-yellow-ochre, turquoise and bluish round scales: a) one subbasal spot between stria II and III, b) one median interrupted stripe from suture towards but not reaching lateral margin, c) two subapical spots on dorsolateral surface, d) a short longitudinal post-median stripe at stria III, and e) one long stripe along lateral margin from base to apex, interrupted before middle. Post-median, and lateral marginal stripe confluent at the apex. ***Legs*** with moderately clavate femora. Femora black covered with subadpressed bluish piliform scales which tend to get longer towards apex and with yellow-ochre, turquoise and bluish oval scales near apical margin. Tibiae covered with suberect long white setae, weakly serrate along inner edge with few protruding teeth. Fore tibiae and midtibiae bear a mucro at apex. Tarsomeres pubescent. Forecoxae covered with colored piliform scales and with pale-yellow-ochre to bluish round scales; mesocoxae and metacoxae covered with setae. Mesoventrite covered with light-colored setae and with light-yellow and turquoise rounds scales on distal ends. Metaventrite densely covered with white setae and with light-yellow ochre and turquoise elliptical scales on distal ends. Ventrite I slightly depressed on disc, densely covered with white setae and with light-yellow to turquoise elliptical scales towards lateral margin. Ventrite II to V sparsely covered with whitish setae. Ventrite V flattened, apical half finely densely punctured, with minute setae.

Male aedeagus as shown in Figs 9–11.

**Female.** Dimensions: LB: 11.8–13.0 mm: LR: 2.3–2.5 mm: WR: 1.9–2.0 mm. LP: 3.5–4.0 mm. WP: 4.0–4.6 mm. LE: 8.0–9.0 mm. WE: 6.0–7.0 mm. *N* = 5.

Habitus as shown in Figs 2 and 4.

Females differ from males in the following: a) pronotum slightly wider, and longer than in male; b) base of pronotum slightly widened on sides, c) elytra longer and moderately wider, lateral contour widest before the middle; and d) ventrite I slightly convex on disc. Otherwise, the female is similar to the male.

#### Etymology.

The new species is named after its type locality, Mt. Melibengoy, which is the local name of Mt. Parker.

#### Distribution.

Metapocyrtus (Orthocyrtus) melibengoy sp. nov. is known from Tboli Municipality, South Cotabato.

### ﻿ Metapocyrtus (Orthocyrtus) flomlok

Taxon classificationAnimaliaColeopteraCurculionidae

Cabras & Medina sp. nov.

52449CE2-7793-5BD3-9407-48787934E5EF

https://zoobank.org/184E0A42-5491-49CC-9B9B-BBE85A79CBE1

Figs 5–8

#### Type material.

**Figures 5–8.**
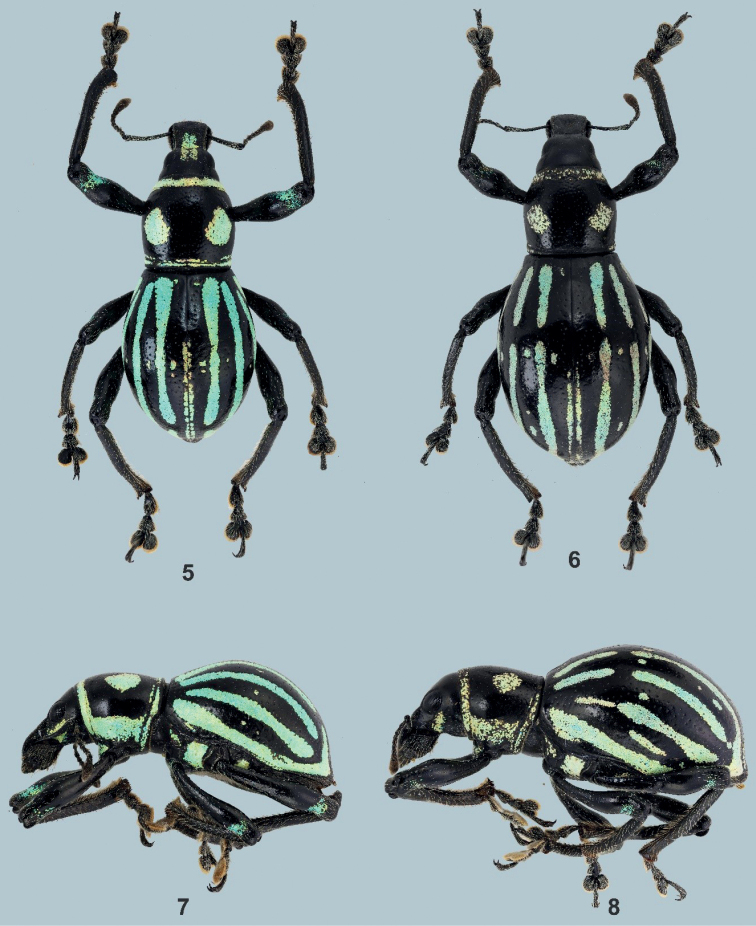
Metapocyrtus (Orthocyrtus) flomlok sp. nov. **5** male holotype, dorsal view **6** female, dorsal view **7** male, lateral view **8** female, lateral view.

***Holotype*** (Figs 5, 7), male: Philippines - Mindanao / Polomolok / South Cotabato / October, 2021 / coll. Cabras (typed on white card) // HOLOTYPE male / Metapocyrtus (Orthocyrtus) flomlok / CABRAS & MEDINA, 2021 (typed on red card). Presently in UMCRC, will be deposited in National Museum of Natural History (PNMNH) under the National Museum of the Philippines. ***Paratypes*** (2♂♂, 2♀♀): same data as holotype; all in UMCRC. All paratypes with additional red label: PARATYPE / Metapocyrtus (Orthocyrtus) flomlok / CABRAS & MEDINA, 2021.

#### Diagnosis.

Metapocyrtus (Orthocyrtus) flomlok sp. nov. is closely related to Metapocyrtus (Orthocyrtus) lanusinus Schultze, 1922 but differs in the following: shorter and stouter body, pronotal scaly marks of two huge round spots on each side of disc, distinct and continuous thick longitudinal stripes from the base to the apex of the elytra, and the stouter and shorter aedeagal body. Meanwhile, M. (O.) lanusinus has a thin transverse band at mid-length, and elytral marks having four interrupted longitudinal stripes distinctly short and oftentimes with short spots in between each longitudinal stripe in the mid-length.

#### Description.

**Male.** Dimensions: LB: 11.0–11.5 mm (holotype 11.0 mm). LR: 2.1–2.3 mm (2.1 mm). WR: 1.8–2.0 mm (1.8 mm). LP: 3.8–4.0 mm (3.8 mm). WP: 4.4–4.7 mm (4.4 mm). LE: 7.0–7.4 mm (7.0 mm). WE: 6.0–6.5 mm (6.0 mm). *N* = 3.

Integument black. Body surface, rostrum, head, and underside with weak luster. ***Head*** finely punctured on dorsum with sparse and very minute setae, frons covered with metallic golden orange, round scales, lateroventral parts below the eye with a semi-elliptical scaly patch of pale-yellow and turquoise round scales, latero-ventral parts with adpressed metallic bluish piliform scales, forehead between eyes nearly flattish. Eyes medium-sized and feebly convex. ***Rostrum*** coarsely rugose on basal 2/3 and finely punctured on apical third, slightly longer than wide (LR/WR:2.1/1.8 mm), dorsum with sparse and adpressed brownish setae, lateral surface with sparse minute subadpressed bluish piliform scales interspersed with brownish and whitish, long suberect setae especially towards the apical margin, ventral surface with long suberect whitish setae; transverse basal groove distinct; longitudinal groove along midline distinct and forms a shallow concavity filled with metallic golden orange with a tinge of green, round scales; dorsum almost flattish dorsally and apex weakly convex; lateral sides with strongly expanded apicad. Antennal scape and funicle of almost the same length, scape reaching the hind margin of eye, covered with subadpressed metallic fine light-colored hairs, and funicle with suberect brownish hairs. Funicular segments I and II almost of the same length, three times longer than wide; segments III–VII nearly as long as wide; club sub-ellipsoidal, nearly 3 times longer than wide. ***Prothorax*** subglobular, wider than long (LP/WP:3.8/4.4 mm), finely punctured especially near anterior margin, widest before middle, weakly convex on dorsal surface, dorsal contour highest point before the middle. Prothorax with the following scaly markings of metallic, light-yellow and turquoise round scales: a) thin stripe at the anterior margin, b) two large subcircular spots on each side of disc, c) thin stripe at the posterior margin, and d) slightly broader stripe before the coxa confluent with the anterior and posterior marginal bands. ***Elytra*** ovate (LE/WE:7.0/6.0 mm), slightly wider and moderately longer than prothorax (WE/WP: 6.0/4.4 mm, LE/LP: 7.0/3.8 mm), finely and distinctly punctured with very minute pubescence, strongly convex, dorsal contour highest before the middle, lateral contour evenly arcuate, widest at middle, apex rounded with sparse, colored, fine setae. Each elytron with the following scaly markings of pale-yellow-ochre, turquoise and bluish round scales: a) three continuous longitudinal scaly stripes from basal margin towards apex of the elytron, b) one long stripe along lateral margin from base to apex, c) one premedian longitudinal stripe along suture, and d) very minute and at times negligible spots in the midle of each scaly stripe at the median portion. ***Legs*** with moderately clavate femora. Femora black covered with subadpressed light blue and turquoise piliform scales and turquoise elliptical scales near the apical margin. Tibiae covered with suberect long white setae, weakly serrate along inner edge with few protruding teeth. Fore and midtibiae bear a mucro at apex. Tarsomeres pubescent. Forecoxae covered with colored piliform scales and with turquoise elliptical scales; mesocoxae and metacoxae covered with setae. Mesoventrite covered with light-colored setae and with turquoise rounds scales on distal ends. Metaventrite sparsely covered with light-colored piliform scales and with turquoise round scales on distal ends. Ventrite I slightly depressed on disc, covered with light-colored piliform scales and with light-yellow to turquoise round scales towards lateral margin. Ventrite II to V sparsely covered with whitish setae and piliform scales which tends to get denser at distal ends. Ventrite V flattened, apical half finely coarsely rugose, with minute setae. Male aedeagus as shown in Figs 12–14.

**Female.** Dimensions: LB: 12.0–12.7 mm: LR: 2.0–2.1 mm: WR: 1.7–1.8 mm. LP: 3.6–3.8 mm. WP: 3.6–3.8 mm. LE: 7.8–8.0 mm. WE: 7.2–7.5 mm. *N* = 2.

Habitus as shown in Figs 6 and 8. Females differ from males in the following: a) base of pronotum slightly widened on sides, b) elytra longer and moderately wider, lateral contour widest before the middle, c) the three stripes from base to apex in the elytra are interrupted before the middle, and d) ventrite I slightly convex on disc. Otherwise female similar to the male.

#### Etymology.

The new species is named after “*flomlok*” the old B’laan name of its type locality Polomolok. The term *flomlok* means hunting ground due to the abundance of wildlife in the area prior to the settlement of lowlanders and agricultural companies.

#### Distribution.

Metapocyrtus (Orthocyrtus) flomlok sp. nov. is known from Polomolok Municipality, South Cotabato.

### ﻿ Metapocyrtus (Orthocyrtus) willietorresi

Taxon classificationAnimaliaColeopteraCurculionidae

Cabras & Medina, 2019

53C1AD8C-F105-55EF-9AD4-FF93E58F6E9E

Metapocyrtus (Artapocyrtus) willietorresi Cabras & Medina, 2019: 186

#### Type locality.

Mt. Apo Natural Park, Davao del Sur.

#### Type depository.

UMCRC.

#### Material examined.

Male: Philippines - Mindanao / Kapatagan / Davao del Sur / December, 2021 / coll. LC (typed on white card). Presently in UMCRC.

#### Remarks.

Cabras and Medina (2019) placed the species under the subgenus Artapocyrtus. However, based on additional materials and further evidence, the authors obviously made some errors, and the species should be placed in the subgenus Orthocyrtus based on the characters mentioned by Cabras et al. (2018). In addition, Cabras and Medina (2019) described the species based on two specimens from Mt. Apo and declared it a new species due to the uniqueness of its elytral and pronotal markings consisting of circular patterns. The shape of the aedeagus of the newly acquired materials (Figs 15–17) further confirms membership of the species in *Orthocyrtus* and its relationship to the *Orthocyrtuslanusinus* species group. One of the defining characteristics of *Orthocyrtus* is the shape of its rostrum. Figs 18–21 show the dorsal view of the different species of *Orthocyrtus* mentioned in this paper.

### ﻿ Metapocyrtus (Orthocyrtus) villalobosae

Taxon classificationAnimaliaColeopteraCurculionidae

Patano, Amoroso, Mohagan, Guiang & Yap, 2021

19077BF0-5917-5D2F-8982-01F504E67EF1

Metapocyrtus (Artapocyrtus) villalobosae Patano et al., 2021: 284

#### Type locality.

Mount Kabunulan, Hamiguitan Range, Surop, Governor Generoso, Davao Oriental, 6°27'44.29"N, 126°10'18.15"E, 400 m a.s.l.

#### Type depository.

CMUZS.

#### Material examined.

4♂♂, 2♀♀: Philippines - Mindanao / San Isidro / Oriental / December, 2021 / coll. LC (typed on white card). Presently in UMCRC.

#### Remarks.

Patano et al. (2021) recently described a new species Metapocyrtus (Artapocyrtus) villalobosae under the subgenus Artapocyrtus based on four specimens collected at Mount Kabunulan, Hamiguitan Range in Governor Generso, Davao Oriental at an elevation of 400 m. Based on the images of the male and female habitus described in the original paper (Patano et al. 2021: 285, fig. 3) with a size ranging from 13.2–14.5 mm and a holotype of 14.5 mm, it doubtlessly belongs to *Orthocyrtus* based on its size, shape of the rostrum, as well as the general shape of the male and female habitus. In 2021, specimens that appeared to belong to Metapocyrtus (Artapocyrtus) villalobosae from San Isidro, Davao Oriental, which is part of the range of Mt. Hamiguitan, were donated to UMCRC. Upon examination, the said specimens were found to be very similar to the male holotype, and female paratype in the paper of Patano et al. (2021). The only difference is the short longitudinal subbasal stripes which we believe are simply part of the variability of the species. We believe it was wrongly placed in the subgenus Artapocyrtus and thus, we are proposing that it be transferred to the subgenus Orthocyrtus based on the characters mentioned by Cabras et al. (2018) including the shape of its rostrum (Fig. 21). Furthermore, while the species elytral patterns may have a superficial resemblance to M. (Sclerocyrtus) chamissoi Schultze, 1925, it is actually more closely related to M. (Orthocyrtus) davaoensis Cabras, Medina & Bollino, 2021 described from Davao City and Bukidnon. The general habitus of the male and female, the elytral and pronotal patterns as well as the genitalia are also very similar. Molecular data and/or eversion of the endophallus in the near future can be used in delineating the *davaoensis* species group which seems to have a widespread form and distribution throughout Mindanao. Eversion of the endophallus in the tribe Pachyrhynchini is quite challenging to accomplish and needs alot of specimens as mentioned by Bollino and Sandel (2017) and Cabras et al. (2018).

**Figures 9–17.**
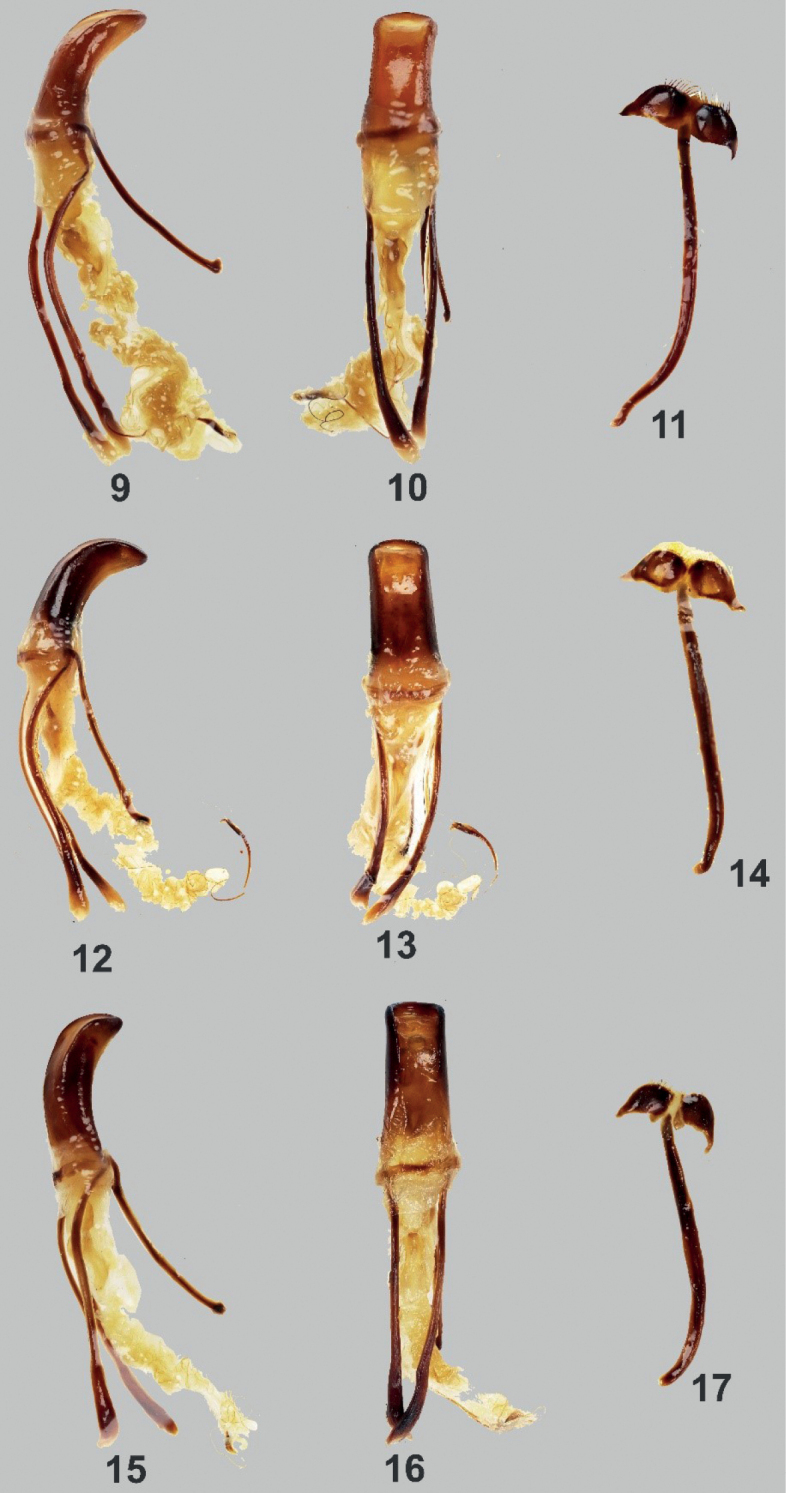
Male genitalia of *Orthocyrtus* sp. **9–11**Metapocyrtus (Orthocyrtus) melibengoy sp. nov. **12–14**Metapocyrtus (Orthocyrtus) flomlok sp. nov. **15–17**Metapocyrtus (Orthocyrtus) willietorresi**9, 12, 15** aedeagus in lateral view **10, 13, 16** idem in dorsal view **11, 14, 17** sternite IX in dorsal view.

**Figures 18–21.**
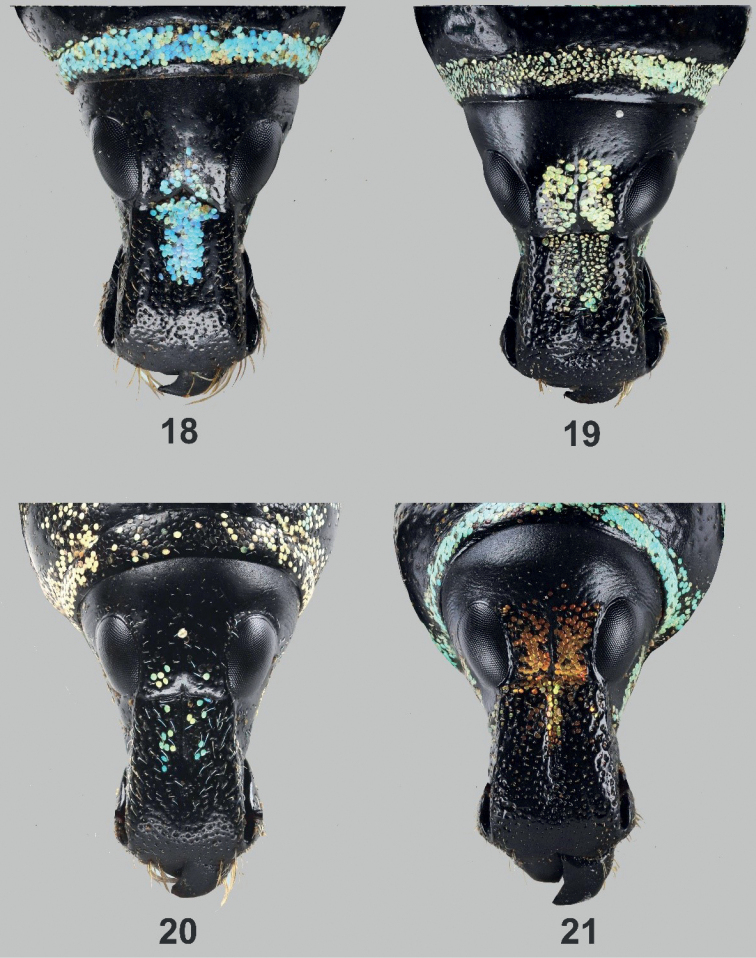
Dorsal view of *Orthocyrtus* spp. rostrums **18**M. (O.) melibengoy sp. nov. **19**M. (O.) flomlok sp. nov. **20**M. (O.) willietorresi**21**M. (O.) villalobosae.

##### ﻿Key to species of Metapocyrtus (Orthocyrtus)

**Table:** 

1	Pronotum coarsely punctured with a transverse scaly stripe in the entire width in the middle; elytra coarsely striate punctate, striae beset with golden yellow and reddish scales	***M* . (*O* .) *villalobosae*Patano et al. 2021**
–	Pronotum subglabrous or finely punctured without a transverse stripe; elytra finely punctate, striae with fine setae	**2**
2	Prothorax as long as wide with subcircular scaly rings on each side of disc; each elytron with two circular scaly rings on basal, medial and apical parts	***M* . (*O* .) *willietorresi* Cabras & Medina, 2018**
–	Prothorax wider than long with scaly spot on each side of disc; elytra with spots or longitudinal scaly stripes from basal margin towards apex	**3**
3	Pronotum with small scaly spots on each side of disc; elytra ovate with subbasal and subapical spots and an interrupted median stripe and a short longitudinal post-median stripe at stria III	***M* . (*O*) . *melibengoy* sp.nov.**
–	Pronotum with large round scaly spot on each side of disc; elytra strongly ovate with thick longitudinal scaly stripes from basal margin towards apex………..	***M* . (*O*) . *flomlok* sp.nov.**

##### ﻿Notes on the habitat of Metapocyrtus (Orthocyrtus)melibengoy sp. nov. and Metapocyrtus (Orthocyrtus)flomlok sp. nov.

**Figures 22–23.**
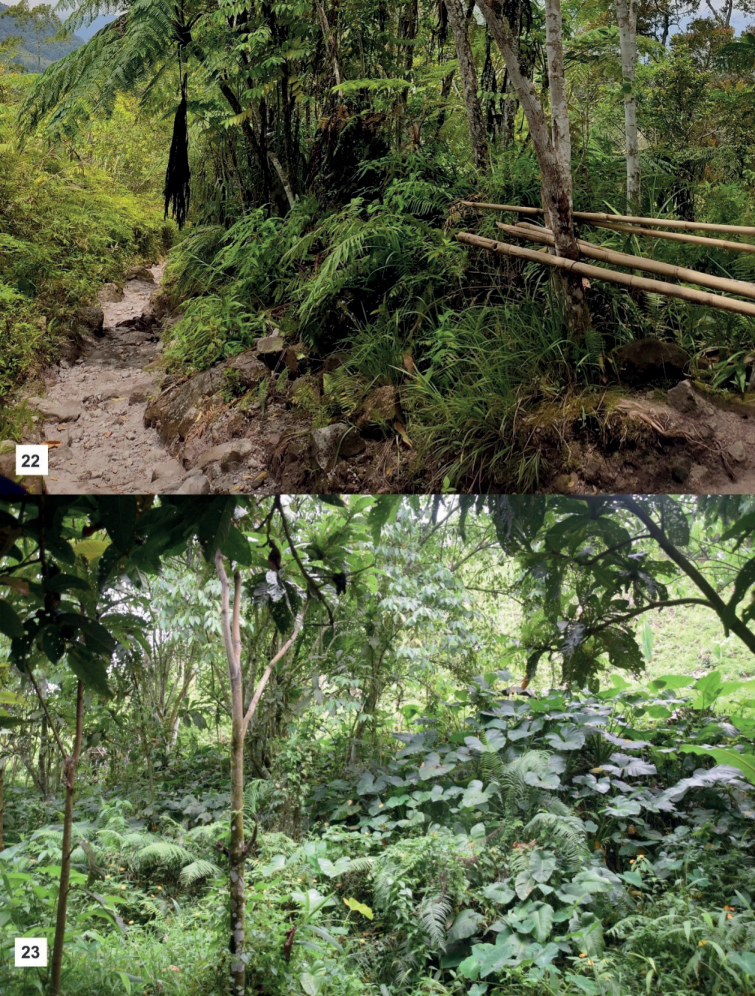
Habitats of *Orthocyrtus* spp. **22**M. (O.) melibengoy sp. nov. in Mt. Parker, T’boli, South Cotabato **23**M. (O.) flomlok sp. nov. in Polomolok, South Cotabato.

Metapocyrtus (Orthocyrtus) melibengoy sp. nov. was collected from forest vegetation along the Salacafe trail leading to Lake Holon, at an elevation of 1200 m (Fig. 22). Lake Holon is a caldera of Mt. Parker, a potentially active stratovolcano in southern Mindanao. The specimens were collected on leaves of a *Melastoma* sp. (Melastomataceae), *Piperaduncum* (Piperaceae), and *Cyathea* spp. (Cyatheaceae) in a partially open area of the trail. The area where the specimens were collected, roughly 9 km from the Barangay Salacafe, serves as the receiving area. The trail to Lake Holon is quite open, with various species of ferns, grasses, and shrubs, and an abundance of pitcher plants and other angiosperms. On the initial ascent to the lake, the trail is quite open with some portions having been converted into farmlands planted with *Zeamays* ssp. Mays (Poaceae), *Coffea* spp. (Rubiaceae), and *Musatextilis* (Musaceae).

As for Metapocyrtus (Orthocyrtus) flomlok sp. nov., it was collected in an open and quite degraded area in Polomolok, South Cotabato, near a pineapple plantation at an elevation of 1021 m. The new species was collected on a slope near a small creek with pristine waters (Fig. 23). It was found on the leaves of buyo-buyo (*Piperaduncum*) and avocado (*Perseaamericana*). During personal correspondence with Stan Cabigas, he mentioned and showed photos of the same species from Mt. Matutum together with its possible model/mimic - *Pachyrhynchusgilvomaculatus* Yoshitake, 2016. Thus, we believe that the population near the pineapple plantation is the remnant population that survived the degradation of the forested habitats and conversion of land in Polomolok. A similar scenario has been observed with M. (Orthocyrtus) davaoensis described based on a limited number of specimens near the lowland of Carmen, Davao City. In our recent surveys of the higher elevation and more forested part of Carmen, a huge thriving population of M. (O.) davaoensis was found.

## ﻿Discussion

The genus *Metapocyrtus* is one of the most taxonomically complex genera of the tribe Pachyrhynchini. The genus is characterized by mimicry with other members of Pachyrhynchini (i.e., *Pachyrhynchus* Germar, 1824, *Trichomacrocyrtus* Yoshitake, 2018, *Eumacrocyrtus* Schultze, 1923) as well as other weevil groups (i.e., *Alcidodes* Marshall, 1939, *Eupyrgops* Berg, 1898, *Polycatus* Heller, 1912, *Calidiopsis* Heller, 1913), and even with the family Cerambycidae as exemplified by the genus *Doliops* Waterhouse, 1841. For the genus *Metapocyrtus*, elytral patterns alone or color variation of the scaly markings should not be used as a basis in delineating species since intersubgeneric mimicry among its members as well as color polymorphism is quite a common occurrence (Schultze 1925; Cabras et al. 2021a). In addition, the shape and general profile of the rostrum, pronotum, and body should be considered in identifying species and not only the elytral patterns. Two almost identical species, in terms of elytral patterns or coloration, could turn out to be two species from different subgenera or even tribes. Such mistakes seem common in the Philippines as evidenced by several online materials as well as specimens housed in some museums, and collections that are erroneously identified.

Another problem with this genus is the subgeneric delineation which remains unresolved and requires a thorough revision. As for the subgenus Orthocyrtus, the large body size, shape of its rostrum, and body make this taxon quite straightforward to identify. Despite recent publications on this subgenus, many species remain unknown and some species may even be cryptic; only eversion of the endophallus or molecular data could help in species delineation. However, because of the unabated loss of forest cover in the Philippines due to illegal logging, conversion of forest lands for commercial or agricultural purposes, as well as mining activities, these species are at a high risk of extinction. Thus, discoveries of new species and research on species biology, ecology, and threats are very important as they may provide the International Union of Conservation of Nature Red List and local stakeholders with evidence to be used for their assessment and conservation initiatives.

## Supplementary Material

XML Treatment for Metapocyrtus (Orthocyrtus) melibengoy

XML Treatment for Metapocyrtus (Orthocyrtus) flomlok

XML Treatment for Metapocyrtus (Orthocyrtus) willietorresi

XML Treatment for Metapocyrtus (Orthocyrtus) villalobosae
